# Inhibition of the thioredoxin system by PX-12 (1-methylpropyl 2-imidazolyl disulfide) impedes HIV-1 infection in TZM-bl cells

**DOI:** 10.1038/s41598-019-42068-2

**Published:** 2019-04-04

**Authors:** Mathias Lundberg, Åse Mattsson, Kathrin Reiser, Arne Holmgren, Sophie Curbo

**Affiliations:** 1 Department of Clinical Science and Education, Södersjukhuset, Internal medicine, Karolinska Institutet, Stockholm, Sweden; 2 Centre for Psychiatry Research, Department of Clinical Neuroscience, Karolinska Institutet, Stockholm, Sweden; 3 Department of Medical Biochemistry and Biophysics, Karolinska Institutet, Stockholm, Sweden; 4 Department of Laboratory Medicine, Division of Clinical Microbiology, Karolinska Institutet, Stockholm, Sweden

## Abstract

Human immunodeficiency virus (HIV-1) entry is initiated by the binding between the viral envelope glycoprotein gp120 and the host receptor CD4, and followed by reduction of structural disulfides of gp120 and CD4. The host thioredoxin-1 (Trx1) efficiently reduces disulfides of gp120 and CD4 *in vitro*, and recently CD4-dependent HIV-1 entry was shown to be inhibited by anti-Trx1-antibodies, indicating a central role for Trx1. 1-methylpropyl-2-imidazolyl disulfide (PX-12) is a reversible inhibitor of the Trx1 system that may also cause a slow irreversible thioalkylation of Trx1. It was developed as an antitumor agent, however, the current study aimed to determine if it also has an anti-HIV-1 effect. We show that PX-12 has anti-HIV-1(III_B_) activity in TZM-bl cells, in fact, no virus was detected inside the cells in the presence of 10 µM PX-12. Moreover, PX-12 inhibited the enzymatic activity of Trx1 and the Trx1-dependent disulfide reduction of gp120. Microtubule polymerization and formation of acetylated microtubules were also inhibited, activities shown to be required for HIV-1 life cycle propagation. In conclusion, our data strengthens the notion that the early steps of the HIV-1 life cycle depends on the Trx1 system and indicate that the Trx1 system may be a rational drug target for HIV-1 treatment.

## Introduction

Human immunodeficiency virus (HIV-1) infection is today successfully treated with combination antiretroviral therapy (cART), using typically three to four anti-HIV-1 drugs^1^. However, due to the emergency of drug resistance and severe side effects, there is still a concurrent need for novel drugs^2^. Viral inhibitors targeting the HIV-life cycle steps, before viral integration (e.g. entry and fusion inhibitors), are among the most attractive targets due to their putative activity to limit *de novo* infected cells. Despite this advantage, the number of inhibitors targeting viral entry and fusion, in clinical use, is limited. The entry of HIV-1 into host cells is initiated by the binding between HIV-1 envelope (composed of a non-covalent complex of three heterodimers of glycoprotein 120 (gp120) and gp41 fusion peptide) and the surface receptor CD4^3^. The receptor interaction causes gp120 and gp41 to undergo major conformational changes, enabling the exposure of binding regions in gp120 to the chemokine co-receptors CXCR4 or CCR5^3^. Subsequently, gp120 and a co-receptor induce gp41 to anchor into the host cell membrane initiating viral membrane fusion and ultimately the release of the viral core into the cytosol. Both gp120 and the host CD4 receptor have been shown to induce acetylation of alpha-tubulin to stabilize the microtubule filaments necessary for fusion of the virus to the cell^4^. For this and subsequent propagation steps, HIV-1 hijacks the cytoskeleton to facilitate entry, uncoating, transport and finally egress from the host cell^5^.

A large body of evidence has indicated that HIV-1 entry is dependent on redox control (reviewed in^6^). The large conformational change of gp120, during viral entry, has been shown to be dependent on the reduction of structural disulfide bonds in gp120^7–9^. Moreover, studies have indicated that the redox status of the structural disulfides of CD4 (i.e. in dithiol or disulfide form) have an essential role for the binding activity of CD4 to gp120^10–13^. Agents that inhibit thiol/disulfide exchange reactions have been shown to suppress HIV-1 viral entry suggesting that redox may be a rational target for HIV-1 treatment^8,9,12–16^. The original studies indicated that protein disulfide isomerase (PDI), a member of the thioredoxin (trx) family, was the responsible catalyst of gp120 disulfide reduction^7–9,16,17^. Agents that specifically suppressed the PDI activity, e.g. anti-PDI antibodies or chemical PDI inhibitors, were shown to inhibit up to 80% of HIV-1 replication and to inhibit viral entry in cultured cells^7–9,16,17^. Later Davies *et al*. showed evidence that also the host oxidoreductase glutaredoxin 1 (Grx1), a member of the trx family, may be involved in the life cycle of HIV-1 by regulating/maintaining the HIV-1 protease activity by a redox mechanism^18^. We subsequently showed that Grx1  is an efficient disulfide reductase for both gp120 and CD4 *in vitro*^14^. Moreover, when the Grx1 activity was inhibited, using specific anti-Grx1 antibodies, >50% decrease in HIV-1 expression was observed, indicating a central role for Grx1^14^. Recent studies by us and others have shown that the host Trx1 is also a very efficient reductase of disulfides of gp120 and CD4 *in vitro*^12,14,15,19,20^. However, the role of Trx1 in the HIV-1 life cycle has shown great complexity, involving a both promoting and inhibitory activity for Trx1^13,15,21,22^. Interestingly, a recent study indicated that specific inhibition of Trx1, using anti-Trx1 antibodies, inhibited HIV-1 entry by >80%, suggesting that Trx1 may have a central role in HIV-1 entry^13^. Trx1 plays a major role in keeping the intracellular *milieu* in a reduced state and, by redox control, the activity of growth factors, coagulation factors, receptors and cytoskeletal proteins including microtubule (MT) and actin^23,24^.

PX-12 (1-methylpropyl 2 imidazol disulfide) was initially described in 1994 as a reversible inhibitor of human thioredoxin reductase but not an inhibitor of glutathione reductase^25^. Later PX-12, then called IV-2, was shown to be a competitive inhibitor in the reduction of Trx1 with a Ki-value of 31 µM^26^. In addition, PX-12 caused reversible inactivation (oxidation) of the active site -Cys-Gly-Pro-Cys- in Trx1 and a slow irreversible inactivation of Trx1 by thioalkylation of Cys 73, a residue in proximity to the active site of Trx1. Thus, PX-12 has broad effects by inhibiting both TrxR1 and Trx1 as a disulfide reductase as well as inactivating Trx1 by irreversibel thioalkylation which blocks its activity as a disulfide reductase of proteins. However, it is not known whether PX-12 has an effect by interfering with the life cycle of HIV-1.

In the present study we have tested the Trx1 system specific inhibitor PX-12 for its activity to inhibit HIV-1 infection. We present data showing that PX-12 has a blocking anti-HIV-1 (III_B_) activity in the micromolar range in the TZM-bl cell model. Our data indicate that Trx1 may have a central role in the early steps of the HIV-1 life cycle and that Trx1 may be a rational drug target for HIV-1 treatment.

## Results

### Cytotoxicity of PX-12 in TZM-bl cells

**Figure 1:**
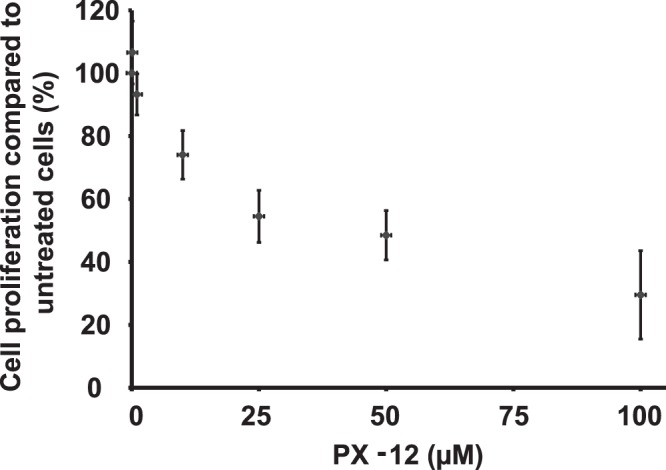
Cytotoxicity of PX-12 in TZM-bl cells. Logarithmically growing TZM-bl cells were exposed to indicate concentrations of PX-12 in the absence of FBS for 3.5 hours, then the medium was replaced with complete medium without PX-12 and the cells were further grown for 48 hours before analyzed by XTT-assay. Data are based on three different experiments and shown as mean ± SEM.

The cytotoxicity of PX-12 on TZM-bl cells was studied by exposing the cells to different concentrations of PX-12 in exactly the same way the cells are exposed to PX-12 in the infectivity assay protocol. The cell proliferation was not much affected in 1 µM PX-12 but reduced by approximately 25% in 10 µM PX-12 (Fig. 1). The inhibitory concentration of 50% (IC_50_) was determined with GraphPad Prism 6 to 40.4 µM (22.8–71.4).

### PX-12 inhibits HIV-1 early infection in TZM-bl cells

**Figure 2:**
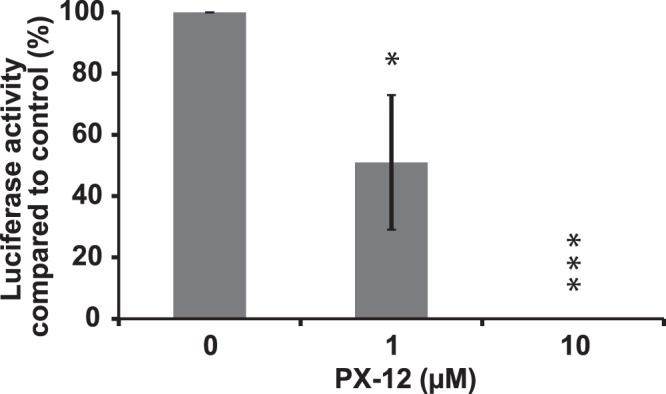
Inhibition of HIV infection in TZM-bl cell cultures. Logarithmically growing TZM-bl cells were infected with HIV-1 in the presence of 0, 1 µM, 10 µM or 100 µM PX-12. After 48 h, the cells were assayed for luciferase activity. The value for the vehicle control was set to 100% in each individual experiment and the negative control was set to 0%. All values were normalized against these reference values. The data are means of three independent experiments and the error bars represent the SEM. *p ≤ 0.05, ***p ≤ 0.01.

In a previous study we observed that organotellurium compounds that interfere with oxidoreductase activity inhibited both HIV-1 and HIV-2 replication by targeting an early event in the viral replication cycle (Reiser *et al*., 2016). PX-12 also affects oxidoreductase activity (Huber *et al*., 2008) and we therefor decided to test if PX-12 interferes with the early infection event by utilizing an infectivity assay consisting of TZM-bl (HeLa/CD4/CXCR4/CCR5) cells which contain a luciferase gene that is under the control of the HIV-1 promoter. In the assay the amount of luminescence generated correlates to the amount of HIV-1 Tat protein inside the cells. PX-12 inhibited the induction of the luminescence signal caused by HIV-1 infection in a dose-dependent fashion (Fig. 2). The presence of 10 µM PX-12 during infection completely inhibited the luciferase activity and even generated a slightly lower luminescence signal than what was detected in non-infected control cells. In cells treated with only 10 µM PX-12 the anti-proliferative effect is about IC_25_ (Fig. 1) and although no visible phenotypic differences were detected under the microscope between control cells and cells treated with up to 10 µM PX-12 (data not shown) the anti-proliferative effect could account for the lower luminescence signal. 1 µM PX-12 did not cause any anti-proliferative effect under the given conditions (Fig. 1) and blocked around 50% of the early infection as determined by the luminescence signal.

### Effect of PX-12 on the enzyme activity of human Trx1, PDI and Grx1

**Figure 3:**
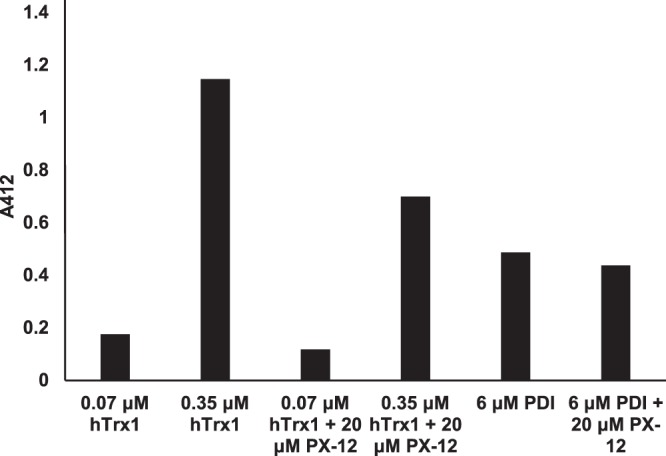
Effect of PX-12 on the enzymatic activity of human Trx1 and PDI. Indicated concentrations of reduced Trx1 or PDI in 50 mM TE buffer and 0.6 mM NADPH was incubated with 20 µM PX-12 for 30 min at 37°C. Then insulin and TrxR were added to a final concentration of 300 µM and 50 nM, respectively, and incubated at 37°C for 20 min. Reaction was stopped by addition of 6 M guanidine-HCl in TE buffer. The absorbance at 412 nm was read against a blank without Trx or PDI. Representative experiment out of several is shown.

A modified insulin reduction assay^27^ using pre-reduced human Trx1 or PDI was used. The proteins were incubated with 20 µM PX-12 for 30 min at 37 °C in the presence of NADPH and then 50 nM TrxR1 and 300 µM insulin was added to start the reaction at 37 °C for 20 min. Reactions were stopped by addition of 6 M guanidine-HCl and DTNB. A typical assay is shown in Fig. 3 where the result of PX-12 is compared to controls. The activity of Trx1 was inhibited whereas no apparent effect was seen for PDI. We tested the effect of PX-12 (20 µM) on the activity of human Grx1 in presence of GSH and fluorescent eosin-bovine serum albumin and no apparent inhibition was observed (data not shown).

### PX-12 inhibits Trx1/TrxR1-mediated gp120 reduction

**Figure 4:**
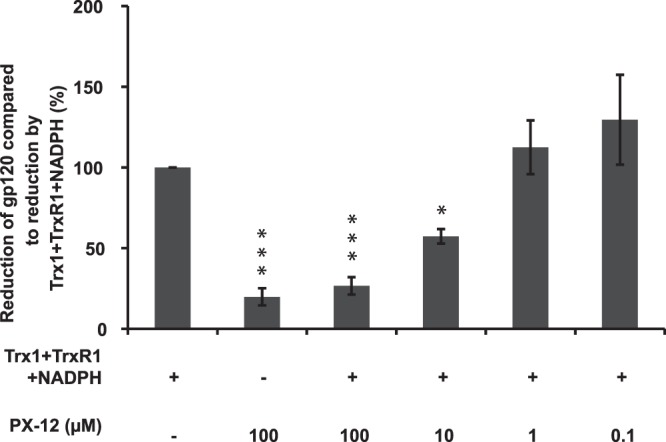
Inhibition of reduction of gp120 in the presence of PX-12. ELISA plates were coated with recombinant human gp120. Reaction mixtures containing 1 µM Trx1 + 100 nM TrxR1 + 240 µM NADPH + (0–100 µM) PX-12 were added to the wells to allow reduction of gp120. After incubation for 15 min the ELISA plates were exposed to streptavidine-ALP and subsequently p-nitrophenyl phosphate and read at 405 nm in a Tecan reader. The value for 1 µM Trx1 + 100 nM TrxR1 + 240 µM NADPH was set to 100% in each individual experiment and all other values were normalized against this reference value. The data are means of three independent experiments; the error bars represent the SEM. *p ≤ 0.05, ***p ≤ 0.01.

HIV binding to CD4 induces disulfide oxidoreduction of gp120 necessary for conformational changes that enable membrane fusion^6^. It has previously been suggested that part of the antiviral effect of different thioredoxin system interfering compounds, including the organotellurium compound TE-2, is due to prevented or decreased gp120 reduction^15,20^. Therefore we tested the ability of PX-12 to inhibit gp120 reduction in a cell-free system. The results show that PX-12 inhibits the thioredoxin system mediated gp120 reduction in a concentration dependent manner (Fig. 4). The strong reducing agent DTT was used a control for breaking up the maximum number of disulfide bonds and compared to the capacity of DTT (when set to 100%) gp120 was reduced to about 60% by the thioredoxin system (Trx1 + TrxR1 + NADPH) (data not shown).

### PX-12 and TE-2 inhibits tubulin polymerization

**Figure 5:**
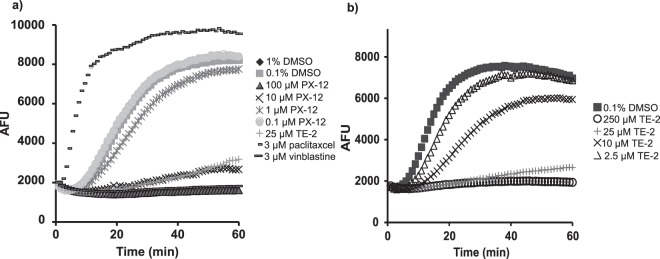
Inhibition of tubulin polymerization in the presence of PX-12 and TE-2. Tubulin polymerization was determined in a cell free system with a tubulin polymerization assay kit from Cytoskeleton. The polymerization of microtubules in the absence/presence of indicated compound was measured as enhancement of fluorescence with data collected with one-minute intervals for 60 min. Tubulin polymerization in the presence of A) 0.1–1% DMSO, 3 µM paclitaxcel, 3 µM vinblastine, 25 µM TE-2 or 0.1–100 µM PX-12 B) 0–250 µM TE-2. All data points were in duplicate and shown as arbitrary fluorescence units (AFU). The experiment was repeated three times but the figure illustrates the results from one representative experiment.

We wanted to test if PX-12 would have an effect in the absence of the Trx1 system and since the cytoskeleton is important for the virus life cycle and it is redox regulated we chose to study tubulin polymerization. It was studied in the presence of different concentrations of PX-12, the depolymerization inhibitor pacliaxel, the polymerization inhibitor vinblastine, the organotellurium compound TE-2 and vehicle controls of 1% and 0.1% DMSO (Fig. 5a). PX-12 has previously been shown to inhibit tubulin polymerization through cysteine oxidation^28^. Here we confirmed that PX-12 inhibits tubulin polymerization in a concentration dependent manner (Fig. 5a). 100 µM PX-12 completely abolished tubulin polymerization in the same magnitude as the polymerization inhibitor vinblastine. 10 µM PX-12 and 25 µM of TE-2 inhibited tubulin polymerization to a similar extent. 100 µM PX-12 was dissolved in 1% DMSO and the same solvent solution was therefore used as a control. There was no effect on tubulin polymerization *in vitro* when treated with 1% or 0.1% DMSO. Neither was there a difference in effect on tubulin polymerization between treatment with water only or water containing up to 1% DMSO (data not shown). Organotellurium compounds have not previously been tested for effect on tubulin polymerization. After determining that 25 µM TE-2 inhibits tubulin polymerization *in vitro* (Fig. 5a) we also tested different concentrations of TE-2 and determined that TE-2 inhibits tubulin polymerization *in vitro* in a concentration dependent fashion (Fig. 5b).

### PX-12 interferes with tubulin acetylation

**Figure 6:**
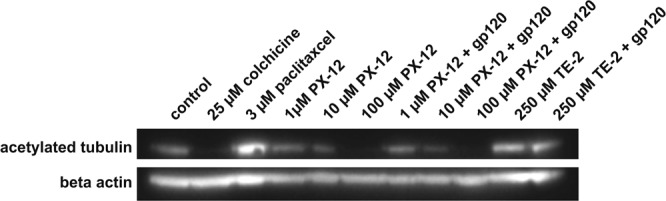
PX-12, but not TE-2, interferes with tubulin acetylation. 80% confluent TZM-bl cells were exposed to 25 µM colchicine (de-acetylating control), 3 µM paclitaxel (acetylating control), medium only (normal control) or 100 µM, 10 µM or 1 µM PX-12 for 30 min before 1 µg/ml gp120 was added to indicated samples and the cells were then further incubated to a total of 18 h. Western blot was performed on the samples with an antibody against acetylated tubulin and an antibody against actin (loading control). The membrane was developed using the SYNGENE system. The experiment was repeated four times and the results shown are representative of the experiments.

Next, we wanted to test if PX-12 would have an effect that was not redox dependent. HIV-1 has been shown to rapidly induce the formation of post-translationally modified stable microtubules that are important for early stages of HIV-infection such as transport of the viral particles to the nucleus^4^. The data from the infectivity assay showed that PX-12 interferes with early HIV-infection events and since tubulin polymerization is affected by PX-12 we decided to test if PX-12 alters tubulin acetylation, a process not solely redox regulated. Results from western blot analysis shows that PX-12 causes tubulin deacetylation in a concentration dependent fashion (Fig. 6). Similar to treatment with 25 µM colchicine, known to cause tubulin de-acetylation, 100 µM PX-12 abolishes the tubulin acetylation completely. 10 µM PX-12 also reduces tubulin acetylation whereas 1 µM PX-12 has no effect. An increase in tubulin acetylation was detected in cells treated with recombinant gp120 to simulate early infection. Incubating the cells with both gp120 and 100 µM or 10 µM PX-12 diminished the acetylating effect of gp120 in the cells to levels similar to cells only exposed to PX-12. 3 µM paclitaxcel was used as a positive control for acetylation. The organotellurium compounds TE-2, −10, −14 and −20 were also tested for inhibition of acetylation (or de-acetylation) of tubulin but no such effect was detected with any of the tested compounds (data not shown). Figure 6 shows cells exposed to 250 µM TE-2 in the presence or absence of gp120 and despite the high concentration used no decrease in tubulin acetylation was detected.

## Discussion

The main objective of this study was to test the anti-HIV-1 activity of the Trx1 system specific inhibitor PX-12. We present data showing that PX-12 has anti-HIV-1 activity, involving a strong inhibition of HIV-1infection. Furthermore, we also show that PX-12 has inhibitory activity on Trx1 and Trx1-dependent disulfide reduction of gp120, the polymerization of microtubules and the formation of acetylated microtubules.

In the presence of 10 µM PX-12 we were unable to detect any intracellular viral Tat-protein. At this concentration of PX-12 we also detected a cytotoxic effect (IC_25_). However, it is unlikely that the cytotoxicity alone could have accounted for the inhibition of HIV infection. This since at a 10-fold lower concentration of PX-12 there was no cytotoxicity and still a 50% inhibition of HIV-infection, supporting a specific antiviral activity of the compound.

Previous studies have shown that PX-12 inhibits both Trx1 and TrxR1 as a disulfide reductase as well as by inactivating Trx1 by irreversible thioalkylation of Cys73 close to its active site^25,26^. The biological role for Trx1, on the HIV-1 life cycle, has been shown to be complex where studies indicate both promotor and inhibitor activity^21,22^. Disulfide/thiol oxidoreduction in at least 7 of the 9 disulfide bridges of gp120 is necessary for virus entry and we and others have shown that Trx1 efficiently catalyze the reduction of gp120, indicating a putative promoting activity on HIV-1 entry^14,19,20^. This assumption was supported by a recent study by Moolla *et al*. that showed that anti-Trx1 antibodies had > 80% inhibitory activity on HIV-1 entry^13^. Our current study suggests that PX-12 also acts as an entry inhibitor although further studies need to be conducted to clarify whether PX-12 mediate its effect with a similar target also in primary cells.Trx1 and TrxR1 are often highly expressed in tumor cells and it is possible that PX-12 only blocks some of the Trx1 system dependent processes in the TZM-bl cells and thereby displays lower toxicity than in primary cells at the same given concentration.

A large body of evidence indicate that redox dependent HIV-1 entry may be catalyzed by two additional members of the trx family, PDI and Grx1^7–9,14,16,17^. To rule out a possible cross-reactivity of PX-12 on PDI and Grx1, we tested the inhibitory activity of PX-12 on Trx1, Grx1, and PDI. As expected, PX-12 inhibited Trx1-dependent activity, although with a rather low activity. However, we were unable to detect a significant inhibitory activity on PDI or Grx1. These data strengthens that the observed effects in the current study are linked to inhibition of Trx1 and further suggests that Trx1 has a central role in early stages of the HIV-1 life cycle. However, an assumption that Trx1 is vital for functional HIV-1 entry is intriguing since previous studies have shown that PDI and Grx1 may play vital roles for HIV-1 entry^7–9,14,16,17^. Altogether the data suggest that functional HIV-1 entry may not depend on an alternative involvement of Trx1, Grx1, or PDI but rather that all these reductases act in concert, as proposed for reductive activity in the endoplasmic reticulum^29^.

When the HIV virus has entered a cell it will activate DNA synthesis by using ribonucleotide reductase to make deoxyribonucleotides from ribonucleotides for reverse transcriptase activity followed by the proviral integration into the DNA genome of the host. If the host cell is a T-cell the Trx1 system is implicated as essential for the T-cell metabolic reprogramming and proliferation^30^. Thus blocking the Trx1 system may have two effects, namely preventing the disulfide reduction in gp120 for entry of the virus as well as preventing the DNA synthesis for proviral synthesis.

Although the initial and primary function of PX-12 as an anti-HIV agent is through inhibition of HIV entry via blocking of the Trx1 system, PX-12 may have effects separted from the Trx1 activity. Since the cytoskeleton is both redox-regulated and important for the HIV-1 life cycle propagation we decided to study tubulin polymerization in the presence of PX-12. In a previous study where Trx1 was silenced, greater growth inhibition was observed for PX-12, pointing to additional mechanisms of action for PX-12^28^. The same study showed that cells treated with PX-12 contained a higher proportion of unpolymerized tubulin and that PX-12 displayed a dose-dependent inhibition of tubulin polymerization mediated via cysteine^28^. We confirmed here that PX-12 inhibits tubulin polymerization in a dose-dependent manner and in addition we determined that the organotellurium compound TE-2 also inhibits tubulin polymerization in a dose-dependent manner. Since the assay is performed in a cell free environment, thus in the absence of the Trx1 system, it is likely that the mechanism by which the organotellurium compound inhibits tubulin polymerization is via cysteine oxidation of tubulin, similar to the mechanism of tubulin polymerization inhibition of PX-12.

To study if PX-12 would have an effect in a process that is not primarily redox regulated we decided to test if PX-12 alters tubulin acetylation. HIV-1 rapidly induces the formation of post-translationally modified stable microtubules, e.g. via acetylation, important for the early stages of HIV-infection such as for transport of the viral particles to the nucleus^4^. In the current study we found that TZM-bl cells treated with PX-12 or organotellurium compounds (TE-2, −10, −14, −20) exhibited different results on tubulin acetylation. In high concentrations PX-12 clearly de-acetylated the tubulin, also in cells treated with gp120 to mimic a viral infection, compared to the tubulin in untreated control cells. The observed de-acetylation of tubulin was concentration dependent just as the depolymerization of tubulin in a cell free system. The organotellurium compounds tested, on the other hand, had no effect on tubulin acetylation in any of the concentrations tested. The compounds do thereby not act by the same mechanisms and although they share some anti-viral properties the compounds have different bioavailability. The results show that higher concentrations of PX-12 make the microtubules unstable and could thereby inhibit the fusion of the virus with the host cell. These results point to that PX-12 may exhibit additional modes of action aside from interference with the Trx1 system. Further studies are needed to unravel the complexity of the effects of PX-12 and the clinical relevance thereof.

In conclusion, our data further strengthens the notion that the early steps of the HIV-1 life cycle may depend on the Trx1 system and indicate that the Trx1 system may be a rational drug target for HIV-1 treatment.

## Materials and Methods

### Materials

Biotin-maleimide, colchicine, DTNB (5,5′-Dithiobis(2-nitrobenzoic acid)), insulin, NADPH, paclitaxel (taxol), PDI, p-nitrophenyl phosphate and PX-12 were purchased from Sigma-Aldrich (Stockholm, Sweden). Recombinant HIV-1(III_B_) glycoprotein gp120 (CHO) was purchased from Immuno Diagnostics, Inc. (Woburn, MA) and Trx1,TrxR1 and the FkGRX-01 kit for Grx1 assay were from IMCO Corporation (Stockholm, Sweden). The compounds 6-(butyltelluro)-6-deoxy-β-cyclodextrin (TE-2), bis[4-(*N*,*N*-di(2-carbomethoxyethyl)amino)phenyl]telluride (TE-10), *N*,*N*-dimethyl-4-aminophenyl 3-phenoxypropyl telluride (TE-14) and 3-(butyltelluro)propanesulfonic acid sodium salt (TE-20) were synthesised as described previously^31–33^ dissolved in dimethylsulfoxide (DMSO) at 25 mM and kept at −20 °C until use. The monoclonal anti-acetylated tubulin antibody (catalogue # T7451, clone 6-11B-1, Batch # 036M4856V 1.1 mg/ml) and anti-β-actin (catalogue # A5441, clone AC-15) were both from Sigma-Aldrich (St. Louis, MO). TZM-bl cells were obtained through the NIH AIDS Reagent Program, Division of AIDS, NIAID, NIH, from Dr. John C. Kappes, Dr. Xiaoyun Wu and Tranzyme Inc. and the persistently HIV-1 infected ACH-2 cells were obtained from Dr. Thomas Folks.

### Cell culture

The TZM-bl cells were cultured in Dulbecco’s modified eagle high glucose medium supplemented with GlutaMAX and 1% fetal bovine serum and 1% Penicillin-Streptomycin and the HIV-1-infected ACH-2 cells were cultured in RPMI 1640 medium supplemented with 10% fetal bovine serum and 1% Penicillin-Streptomycin (all reagents from Gibco) and kept at 37 °C and 5% CO_2_.

### Cell proliferation assay

10^4^ TZM-bl cells/well were seeded in 100 µl complete DMEM medium in a 96 well plate. 24 hours later the cells were washed twice with serum free DMEM medium and added serial dilutions of PX-12 diluted in serum free DMEM medium. Negative control cells were added 0.5% (v/v) DMSO, the same concentration of DMSO used in the highest concentration of PX-12, in either complete or serum free DMEM medium. To replicate the conditions used in the infectivity assay the cells were then incubated in the presence or absence of PX-12 at 37 °C and 5% CO_2_ for 1 hour and then added 1 µl serum free DMEM medium/well before incubated for additional 2.5 hours. The cells were then washed twice with complete DMEM medium before each well was added 100 µl complete DMEM medium (without any PX-12) and further incubated 48 hours before assayed with the XTT method (Roche). The data is based on three separate experiments and presented as the inhibitory concentration (IC_50_) defined as the PX-12 concentration required to inhibit cell proliferation by 50% *in vitro*. Acquired data were analyzed by GraphPad Prism 6 and are displayed as IC_50_ and the 95% confidence interval within brackets.

### Virus expression

Virus expression was performed as previously described (Reiser *et al*., 2016) Briefly, persistently HIV-1-infected ACH-2 cells (1 × 10^6^ cells/ml) were cultured in the presence of 100 nM PMA for three days. Then the culture supernatant was collected, subjected to centrifugation at 300 x g for 10 min and passed through a 0.45 µm filter. The HIV p24 content was measured in an Architect i2000SR (Abbott Laboratories, IL) and the virus suspension was either used directly or at −80 °C.

### Infectivity assay

1 × 10^4^ TZM-bl cells/well were seeded in 100 µl complete DMEM medium in a 96 well plate (Nunc). 24 hours later the cells were washed twice with serum-free DMEM medium and then exposed to 0, 0.1 µM, 1 µM or 10 µM PX-12 diluted in serum-free DMEM medium for 1 hour. Negative control cells (0 µM PX-12) were added 0.5% (v/v) DMSO, the same concentration of DMSO used in the highest concentration of PX-12. Subsequently, virus suspension was added to all wells, except wells kept as non-infected controls. The cells were then further incubated for 2.5 hours at 37 °C and 5% CO_2_ before the cells were washed twice and the medium replaced with complete medium. After 48 hours incubation the supernatants were collected for p24 measurements and luminescence was assayed and quantified in a Luminoskan Ascent (Thermo Labsystems, Schwerte, Germany) for luciferase activity using the OneGlo luciferase assay system (Promega, Nacka, Sweden). The HIV p24 content was measured in an Architect i2000SR (Abbott Laboratories, IL). The data is based on four separate experiments. Infection in the absence of PX-12 is set to 100% and the values are presented as mean ± SEM. Two tailed student’s t-test was used to test for statistical significance.

### Effect of PX-12 on the enzymatic activity of Trx1, PDI and Grx1

Human Trx1 and PDI proteins were reduced by incubation with 1 mM DTT for 30 min at 37 °C followed by desalting to TE buffer (50 mM Tris-HCl, 1 mM EDTA, pH 7.5). Using 95 µl of 0.6 mM NADPH in TE buffer an aliquot of reduced Trx1 or PDI was incubated with 20 µM PX-12 for 30 min at 37 °C. Then the insulin disulfide reaction^27^ was started by adding insulin and TrxR1 at final concentrations of 300 µM and 50 nM, respectively, followed by incubation for 20 min at 37 °C. The reaction was stopped by mixing with 500 µl of 6 M guanidine-HCl in TE containing 10 mM DTNB. The absorbance at 412 nm was measured in a 1 cm cuvette to determine formed SH-groups. Controls were run without PX-12. Human Grx1 activity was assayed by using a kit called FkGRX-01 (IMCO) with and without 20 µM PX-12.

### Inhibition of TrxR1/Trx1-dependent reduction of HIV-1 gp120

96-well Maxi-Sorp ELISA plates (Nunc Inc, Roskilde, Denmark) were coated with 100 µl gp120 in 50 mM carbonate buffer pH 9.6 (2 µg/ml) over night at 4 °C and then blocked with PBS-T (phosphate buffered saline, pH 7.4 with 0.05% Tween 20) for 1 h at 37 °C. The experiment was performed with reaction mixtures containing 1 µM Trx1 + 100 nM TrxR1 + 240 µM NADPH + indicated concentrations of PX-12 (0–100 µM), 5 mM dithiothreitol was used as positive control and PBS as a negative control. The reaction mixtures were added to the gp120-coated wells for 30 min at 37 °C and then washed four times with PBS-T to remove any unbound material. Subsequently, the wells were incubated with 10 µM biotin-maleimide for 30 min at room temperature, then washed and incubated with steptavidine-ALP (Mabtech, Nacka, Sweden) diluted 1:1,000 in PBS for 30 min at room temperature. The wells were washed four times with PBS-T and 1 mg/ml p-nitrophenyl phosphate dissolved in 10% diethanolamine pH 9.8 with 0.5 mM MgCl_2_ was added. The absorbance at 405 nm was measured using a microplate reader (Tecan).The data is based on three separate experiments. The value for 1 µM Trx1 + 100 nM TrxR1 + 240 µM NADPH was set to 100% in each individual experiment and all other values were normalized against this reference value. The data shown are means of three independent experiments and the error bars represent the SEM.

### Tubulin polymerization assay

Tubulin polymerization was determined *in vitro* with a tubulin polymerization assay kit (Cytoskeleton, Inc) according to the manufacturer’s protocol. Briefly, the assay plate was pre-warmed to 37 °C before 5 µl of a 10 × stock solution of the drug of interest or control was added to each well. The plate was then incubated at 37 °C for 2 min before 45 µl of a tubulin solution was added to each well. Polymerization of microtubules was then immediately initiated and measured as enhancement of fluorescence. Readings were done on an Infinite M200 (Tecan) using excitation at 360 nm and emission at 450 nm. Data was collected with one-minute intervals for 60 min and analyzed using Magellan software. All data points were in duplicate and shown as arbitrary fluorescence units (AFU). The data is based on three separate experiments.

### Western blot analysis

TZM-bl cells were seeded in 6 well plates (Nunc) and allowed to grow to 80% confluence before treated with 25 µM cholchicin (de-acetylating control), 3 µM paclitaxel (acetylating control) or duplicate samples of medium alone or 100 µM, 10 µM or 1 µM of PX-12 or 25 or 250 µM of TE-2, TE-10, TE-14 or TE-20 for 30 min at 37 °C and 5% CO_2_. Then 1 µg/ml gp120 was added to one of the respective duplicate samples and further incubated at 37 °C and 5% CO_2_ for additional 17.5 h. The cells were then washed with PBS and harvested with RIPA buffer. 10 µg of the cell lysates were loaded onto a 4–12% Bis-Tris gel (Bio-Rad) and transferred onto a PVDF membrane (GE Healthcare). The membrane was blocked over night at 4 °C with 5% bovine serum albumin (Sigma) diluted in PBS, washed three times in PBST (PBS and 0.05% tween 20) and again incubated over night at 4 °C with the anti-acetylated tubulin antibody diluted 1:1,000 in PBS with 5% BSA. The membrane was washed 4 times in PBST and incubated at room temperature for 2 hours with the anti actin antibody diluted 1:5,000 in PBS with 5% BSA. The membrane was washed 4 times with PBST and incubated at room temperature for 2 hours with the HRP conjugated anti-mouse IgG antibody diluted 1:3,000 in PBS with 5% BSA. The membrane was washed 4 times in PBST and then developed using the ECL-system. The membrane was also developed using the SYNGENE system and the intensities of the bands were quantified with the SYNGENE gene tools software. The experiment was repeated four times and the results shown are representative of the experiment.
